# Prediction of Mucosal Health by NLR, CRP x NLR and MPV in Ulcerative Colitis: Can Their Availability Change According to Treatment Options?

**DOI:** 10.7759/cureus.19942

**Published:** 2021-11-27

**Authors:** Sami Cifci, Nergis Ekmen

**Affiliations:** 1 Gastroenterology, Basaksehi Cam and Sakura City Hospital, Istanbul, TUR; 2 Gastroenterology, Gazi University Medical School, Ankara, TUR

**Keywords:** c –reactive protein (crp), ulcerative colitis, neutrophil-lymphocyte ratio, mean platelet volume, ınflammation

## Abstract

Introduction

Mucosal healing is the main treatment goal in ulcerative colitis (UC). Many noninvasive parameters have been used in clinical practice to assess mucosal healing. In this study, we aimed to evaluate the effectiveness of neutrophil-lymphocyte ratio (NLR), C-reactive protein (CRP) x NLR and mean platelet volume (MPV) in predicting mucosal health.

Method

This study was designed as a retrospective and single-center. A total of 165 patients, 126 active and 39 in remission, were included in this study. The patients were divided into two groups. The patients were divided into two groups. Group-1 consisted of newly diagnosed patients and patients using only mesalazine; Group-2 was including patients using CS and/or AZT plus mesalazine for at least one month. The relationship between Rachmilewitz endoscopic activity index (EAI) and erythrocyte sedimentation rate (ESR), CRP, NLR, CRP x NLR, MPV and platelet (PLT) was evaluated. Using receiver operating characteristic curves, cut-off values were determined for these parameters to predict active disease.

Results

A positive correlation was found between CRP, PLT and NLR and EAI (p<0.001). A negative correlation was found between MPV and EAI (p<0.001). The accuracy of CRP, NLR, CRP x NLR and PLT (2.65 mg/dl, 2.06, 4.29 and 278.5 x 10^9^/L at the indicated cut-off values, respectively) in detecting disease activity was 77.0%, 65.1%, 77.0% and 72.2%, respectively. MPV was not statistically significant in predicting disease activation (p> 0.05).

Conclusion

CRP and CRP x NLR are significant non-invasive markers for detecting mucosal health in UC. In addition, these markers can be used to evaluate mucosal health regardless of treatment types.

## Introduction

Ulcerative colitis (UC) is a chronic inflammatory disease of unknown etiology, manifested by erythema and ulceration in the colon. Fever, diarrhea and rectal bleeding are the most important symptoms of acute attack periods. Corticosteroids (CS) are partially effective in inducing remission of UC; however, approximately 20%-30% of cases are resistant or dependent on CS treatment [1]. In such cases, immunomodulators (IMs) are recommended to maintain remission after induction therapy. Classification of UC by disease activity is important for determining disease severity in clinical practice. In practical application, a combination of clinical, laboratory, imaging, histopathological and endoscopic parameters is used to determine the activity of the disease [2]. However, none of these have been identified as an ideal marker on their own.

In patients with ongoing inflammation, increased proinflammatory cytokines, especially interleukin-6, cause platelet (PLT) release, and these PLTs, whose number increases, migrate to the area of inflammation [3]. Damage to these PLTs in the area of inflammation and degranulation of their cytoplasm leads to a decrease in mean platelet volume (MPV) [4]. It has been suggested that low MPV level is associated with inflammatory conditions in many rheumatic diseases and MPV works as a negative marker in these diseases [5]. On the contrary, in some studies conducted in patients with rheumatoid arthritis (RA) and systemic lupus erythematosus, it was reported that the MPV value was significantly higher and correlated with disease activity [6,7]. Data on MPV in Crohn's disease (CD) are not yet clear. In a study, although there was a decrease in MPV compared to healthy controls, it could not distinguish between active and inactive CD patients [8]. In contrast to CD, patients with active UC have been described to have a significantly lower MPV and negatively correlate with the endoscopic activity index (EAI) [9]. Similarly, studies have been conducted to examine disease activity for neutrophil-lymphocyte ratio (NLR) in some inflammatory diseases. It has been reported that NLR is positively correlated with C-reactive protein (CRP), erythrocyte sedimentation rate (ESR), and disease activity score in RA patients [10].

Various non-invasive markers have been used to detect disease activity as an alternative to classical inflammatory markers in UC. However, the efficacy of these markers in patients under treatment is unknown. The effects of IM drugs such as azathioprine (AZT), which are frequently used in the treatment of inflammatory bowel disease (IBD), and CS on peripheral blood cells should not be ignored [11,12]. Based on this, we aimed to evaluate NLR, CRP x NLR and MPV according to disease activity in patients using CS and IM drugs.

## Materials and methods

Ethical approval

The study was approved by the Noninvasive Clinical Research Ethics Committee of Basaksehir Cam and Sakura City Hospital (Decision number: 2021-243, dated: 03.11.2021 ).

Study design

This study was designed as a single-center retrospective study. Patients who were followed up with the diagnosis of UC and diagnosed with UC between June 2020 and December 2021 were retrospectively reviewed from hospital records. The diagnosis of UC was based on clinical, endoscopic and histopathological findings [13]. Demographic, clinical and laboratory data were obtained from medical records and recorded. Patients diagnosed with malignancy and another inflammatory-autoimmune disease were excluded from the study. Patients whose Rachmilewitz EAI was calculated during colonoscopy were included in the study. Patients with EAI ≥ 4 were considered active [14]. The patients were divided into two groups. Group-1 consisted of newly diagnosed patients and patients using only mesalazine; Group-2 was including patients using CS and/or AZT plus mesalazine for at least one month. Patients were classified by the extent of colonic involvement according to the Montreal classification as follows: ulcerative proctitis (E1); left-sided colitis (E2); and extensive colitis (E3) [15].

The sample number calculated using OpenEpi was determined as a minimum of 132, taking the ratio of sample size:3 with 95% confidence interval and 80% power.

Blood samples of the patients were taken into a tube with ethylenediaminetetraacetic acid (EDTA), and hematological analysis of the samples was performed on the XN-900 (SysmexTM, Japan) device. The biochemistry parameters were analyzed in the SF-8200 (RocheTM Cobas 8000, USA) device. NLR value was calculated from blood samples by dividing the absolute neutrophil count by the absolute lymphocyte count. Endoscopies were performed by a gastroenterologist using Fujinon Video Colonoscope EC-580RD-L (Fujifilm TM Europe, Düsseldorf, Germany) colonoscopy devices.

Statistical analysis

The data were analyzed using the Statistical Package for the Social Sciences v22 (SPSS, Inc, Chicago IL, USA). Continuous variables among independent groups were analyzed with the Mann-Whitney U test. Pearson chi-square (χ2) test was used in the comparison analysis for categorical variables among independent groups. In cases where the variables were not normally distributed, a nonparametric test of Spearman correlation was used instead.

Receiver operating characteristic curve (ROC) analysis was performed to calculate the active disease predictive value of NLR, MPV, CRP x NLR. The area under the ROC curve (AUC) results were considered as follows: 0.9-1, excellent; 0.8-0.9, good; 0.7-0.8, fair; 0.6-0.7, poor; and 0.5-0.6, failed. The results following ROC analysis, AUC and cut-off values, sensitivity, and specificity of these cut-off values were presented. p<0.05 was considered statistically significant.

## Results

Evaluation of clinical and laboratory findings according to disease activation

A total of 165 patients, 126 active and 39 in remission, were included in this study. Both groups were similar in terms of age and gender.

In the examination made in terms of disease localization, it was determined that the majority of the patients in remission had left-sided involvement, while the majority of the patients in the active period were found to have extensive involvement. There was a statistically significant difference between the groups in terms of the site of involvement (p<0.001). The results of the comparison of laboratory parameters between active and remission patient groups are summarized in Table 1. According to these results, the CRP level in the activation group was statistically significantly higher than the patients in remission (p<0.001).

**Table 1:** Comparison of clinical and laboratory parameters according to disease activation. Mann-Whitney U test was used. ESR: erythrocyte sedimentation rate; CRP: C-reactive protein; WBC: white blood cell; NLR: neutrophil-lymphocyte ratio; MPV: mean platelet volume. *Median (min-max).

	Active, n=126	Remission, n=39	p
Age, year (mean±SD)	38.56±14.60	43.64 ±13.38	0.733
Gender, n (%)			0.102
Female	54 (42.9)	11 (28.2)	
Male	72 (57.1)	28 (71.8)	
Involvement area, n (%)			<0.001
Ulcerative proctitis	7 (5.6)	4 (10.3)	
Left-sided UC	42(33.3)	26 (66.7)	
Extensive UC	77 (61.1)	9 (23.1)	
*ESR (mm/hr)	32 (5-100)	20 (1-56)	0.004
*CRP (mg/L)	8.95 (0.20-240.68)	1.90 (0.16-18.00)	<0.001
*WBC (x10^9^/L)	9.00 (2.90-26.30)	6.80 (4.10-13.90)	<0.001
*Haemoglobin (g/dL)	12.05 (6.80-15.60)	13.70 (10.20-16.60)	<0.001
*Platelet (x10^9^/L)	341 (164-844)	305 (181-473)	0.015
*Neutrophile (x10^9^/L)	5.60 (1.26-21.00)	3.80 (1.90-12.20)	0.001
*Lymphocyte (x10^9^/L)	2.10 (0.80-4.80)	1.90 (1.20-3.30)	0.670
*NLR	2.42 (1.05-16.00)	2.04 (1-9.38)	0.002
*MPV	9.80 (6.50-9.90)	10.20 (8.50-11.50)	0.211

In the evaluation made according to the type of treatment, the patients were divided into two treatment groups as group-1 (n=119) and group-2 (n=46). In the comparison of laboratory parameters according to these groups, statistically significant difference was found between the groups only in terms of ESR (p=0.002), other biochemical parameters were similar (p>0.05) (Table 2).

**Table 2:** Comparison of serum inflammatory parameters according to treatment type. Mann-Whitney U test was used. ESR: erythrocyte sedimentation rate; CRP: C-reactive protein; WBC: white blood cell; NLR: neutrophil-lymphocyte ratio; MPV: mean platelet volume. *Median (min-max).

	Group-1: Newly diagnosed patients and patients using mesalazine, n=119	Group-2: Patients using CS and/or AZT plus mesalazine, n=46	p
*ESR (mm/hr)	32 (2-95)	16 (1-100)	0.002
*CRP (mg/L)	8 (0.16-240.68)	3.60 (0.20-85.52)	0.236
*WBC (x10^9^/L)	8.40 (2.90-26.30)	7.55 (3.60-15.70)	0.087
*Haemoglobin (g/dL)	12,40 (6.80-16.60)	12.65 (9.10-15.30)	0.913
*Platelet (x10^9^/L)	320 (177-844)	337 (164-491)	0.785
*Neutrophile (x10^9^/L)	5.30 (1.26-21.00)	4.50 (1.69-11.40)	0.053
*Lymphocyte *(x10^9^/L)	2.00 (0.80-4.80)	2.10 (1.10-2.80)	0.891
*NLR	2.37 (1.00-16.00)	1.90 (1.03-10.00)	0.039
*MPV	9.90 (6.50-13.60)	9.75 (8.30-13.40)	0.592

The correlation analysis between Rachmilewitz score and inflammatory parameters is summarized in Table 3. There was a weak positive correlation (r=0.78, p<0.001) between Rachmilewitz score and ESR. A moderate positive correlation was found between CRP, WBC, PLT, NLR, neutrophile and Rachmilewitz score (respectively, r=0.647, p<0.001; r=0.502, p<0.001; r=0.410, p<0.001; r=0.499, p<0.001 and r=0.442, p<0.001). A moderate and weak negative correlation was found between haemoglobin, MPV and Rachmilewitz score (respectively, r=-0.360, p<0.001; r=-0.277, p<0.001). No correlation was found between lymphocyte and Rachmilewitz score (r=0.025, p=0.745).

**Table 3:** Evaluation of the relationship between ulcerative colitis activity score and serum inflammatory parameters. Spearman correlation analysis was used. ESR: erythrocyte sedimentation rate; CRP: C-reactive protein; WBC: white blood cell; NLR: neutrophil-lymphocyte ratio; MPV: mean platelet volume.

	r	p
Rachmilewitz score		
ESR	0.278	<0.001
CRP	0.647	<0.001
WBC (x10^9^/L)	0.502	<0.001
Haemoglobin (g/dL)	-0.360	<0.001
Platelet (x10^9^/L)	0.410	<0.001
Neutrophile (x10^9^/L)	0.499	<0.001
Lymphocyte (x10^9^/L)	0.025	0.745
NLR	0.442	<0.001
MPV	-0.277	<0.001

Analysis of disease activation predictive values ​​of NLR, CRP, PLT, MPV and CRP x NLR

The predictive power of NLR, CRP, PLT, MPV, and CRP x NLR was evaluated by ROC analysis. AUC values and statistical significance obtained from ROC analysis are presented in Table 4. MPV was not statistically significant in predicting disease activation (p>0.05).

**Table 4:** Performance of serum inflammatory markers for prediction disease activation. *Statistically not significant area under the curve (AUC) obtained from the receiver operating characteristic. NLR: neutrophil-lymphocyte ratio; CRP: C-reactive protein; MPV: mean platelet volume.

Variable	AUC (95% CI)	p	Cut-off	Sensitivity	Specificity
NLR	0.629(0.576-0.760)	0.002	≥2.06	65.1	64.1
CRP	0.795(0.724-0.867)	0.037	≥2.65	77.0	64.1
CRP x NLR	0.788 (0.712-0.864)	<0.001	≥4.29	77.0	59.0
*MPV	0.566 (0.467-0.666)	0.211	≤9.95	57.1	61.5
Platelet	0.629 (0.541-0716)	0.047	≥278.50	72.2	46.2

**Figure 1:**
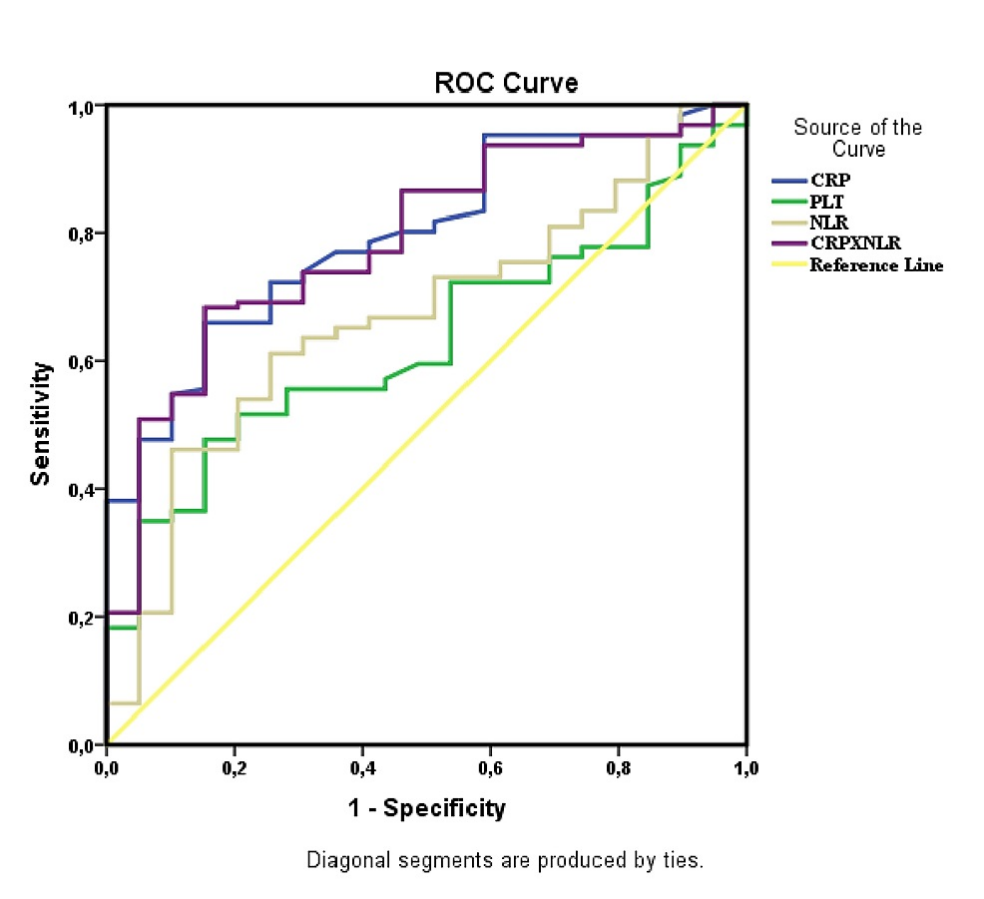
Receiver operating characteristic (ROC) curves for NLR, CRP, PLT vs CRP x NLR NLR: neutrophil-lymphocyte ratio; CRP: C-reactive protein; PLT: platelet.

According to the ROC curve analysis, the sensitivity and specificity of NLR were found to be 65.1% and 64.1% in predicting activation at a cut-off value of 2.06. CRP x NLR had a sensitivity of 77.0% and a specificity of 59.0% in predicting activation at a cut-off value of 4.29. At the 2.65 cut-off value of CRP, the sensitivity was 77.0% and the specificity was 64.1%. At the cut-off value of 278.50, the sensitivity of PLT was 72.2% and the specificity was 46.2% (Figure 1).

## Discussion

Clinical studies have found an increase in blood PLT counts in UC and CD, as well as a significant decrease in MPV and PLT distribution widths [16,17]. In a recent study, it was reported that the number of PLTs was higher in patients with active UC, the rate of MPV was lower, and there was a negative correlation between MPV and disease activity [18]. In the current study, it was found that EAI had a negative correlation with MPV and a positive correlation with PLT, which supported this study. In ROC analysis, however, it was not found to be significant in predicting the disease activity of MPV. This may be due to the relatively small number of active patients and that some of these patients are under treatment. The sensitivity of PLT at a cut-off value of 278.5 x109/L was found to be 72.2%.

In a study evaluating NLR in patients with UC, it was reported that NLR values were higher in active UC compared to those with UC in remission, and the optimum NLR cut-off value for active UC was 2.39 [19]. In another study, a cut-off value of 2.16% showed the presence of active disease with a sensitivity of 81.8% and a specificity of 80.5%, and it was reported that NLR values were correlated with leukocyte and ESR levels [20]. In a study examining the risk of pouchitis development, it was reported that high NLR had a strong relationship with the risk of pouchitis development [21]. In the current study, a positive correlation was found between CRP and NLR and Rachmilewitz EAI. According to ROC curve analysis, the sensitivity and specificity of NLR in predicting activation were 65.1% and 64.1% at a cut-off value of 2.06.

To our best knowledge, it seems that the only study in the literature that evaluated CRP x NLR was on the prediction of acute appendicitis. In this study, the area under the ROC value was 0.83 for NLR, 0.73 for CRP, and 0.81 for CRP x NLR, and it was interpreted that NLR is more useful than CRP [22]. In the current study, the sensitivity of CRP x NLR in predicting activation at a cut-off value of 4.29 was 77.0%, and the specificity was 59.0%. At the cut-off value of CRP 2.65 (mg/dl), the sensitivity was 77.0% and the specificity was 64.1%. Our findings show that NLR alone cannot adequately detect active disease, but CRP x NLR is more useful.

When the patients were divided into those who use CS and/or IM drugs and those who do not, there was a significant difference between the groups only in terms of ESR and it was found to be significantly lower in the group using CS and/or AZT.

The limitations of this study are the low number of active patients, the decrease in the number of patients during classification according to treatment groups, and the retrospective nature of the study.

## Conclusions

In conclusion, the current study shows that the best parameters for mucosal health are CRP and CRP x NLR. Determination of mucosal health by non-invasive methods in UC is of great importance. As a proven parameter, CRP has an important place in determining activation today, and we also think that the usability of CRP x NLR should be evaluated with further studies.
